# DNA interfaces with dimensional materials for biomedical applications

**DOI:** 10.1039/d1ra04917h

**Published:** 2021-08-23

**Authors:** Narges Asefifeyzabadi, Prabhangshu Kumer Das, Avokerie Hillary Onorimuo, Grace Durocher, Mohtashim Hassan Shamsi

**Affiliations:** School of Chemical and Biomolecular Sciences, Southern Illinois University Carbondale IL USA mshamsi@siu.edu +1-618-453-6408 +1-618-453-6461

## Abstract

DNA interfaces with nano, micro, and macro materials have gained widespread attention for various applications. Such interfaces exhibit distinct functions and properties not only due to the unique properties of interfacing materials but also sequence- and conformation-dependent characteristics of the DNA. Therefore, DNA interfaces with diverse dimensional materials have advanced our understanding of the interaction mechanisms and the properties of such interfaces. The unique interfacial properties of such novel materials have applications in nanotechnology, biophysics, cell biology, biosensing, and bioelectronics. The field is growing rapidly with the frequent emergence of new interfaces carrying remarkable interfacial character. In this review article, we have classified the DNA interfaces into 0D, 1D, 2D, and 3D categories based on the types of dimensional materials. We review the key efforts made in the last five years and focus on types of interfaces, interfacing mechanisms, and their state-of-the-art applications. This review will draw a general interest because of the diversity in the DNA materials science but also the unique applications that will play a cutting-edge role in biomedical and biosensing research.

## Abstract 2

DNA interfaces with 0–3 dimensional materials through physisorption and chemisorption mechanisms acquire unique interfacial character for novel biomedical applications.

## Graphical abstract

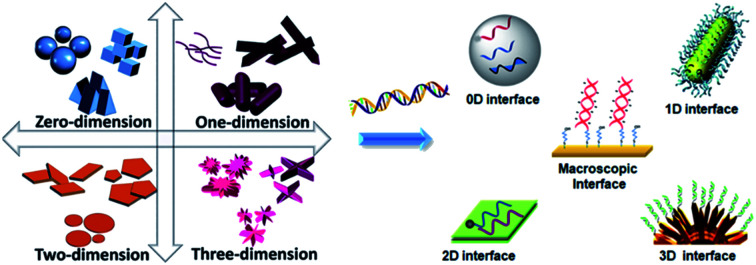

## 1 Introduction

**Fig. 1:**
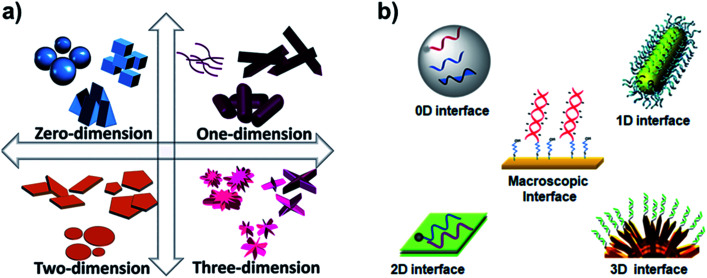
(a) Types of dimensional materials. Adapted from IntechOpen, DOI: 10.5772/intechopen.83803. (b) Types of DNA interfaces with dimensional materials.

DNA interactions with metals and their ions were the focus in the past for their change in structural proeprties and applications.^1^ However, DNA interfaces with diverse dimensional materials (*i.e.* 0D, 1D, 2D, and 3D), Fig. 1a, are rapidly growing having diverse interaction mechanisms, nucleic acid behavior, and interfacial properties. The DNA–dimensional materials interfaces have wide applications in nanotechnology, biophysics, cell biology, biosensing, bioelectronics biomedical research.^2–5^ DNA interfaces with dimensional materials, Fig. 1b, demonstrate distinct physical and chemical properties that can be promising for signal reporting, transduction mechanism, and amplification for a wide range of biological applications.^6–9^ Interfacing mechanisms have an important role in achieving desired properties for such materials.^10^ The optical properties (*e.g.* absorbance, reflectance, scattering, plasmon resonance, and luminescence) of the designed materials can be monitored for optical diagnostic systems.^11^ Electrical and electrochemical techniques monitor charge transport at the DNA–materials interfaces, which can be transformed into simple and miniaturized platforms with high sensitivity.^12,13^ Surface properties of the interfaces through surface probe microscopy can facilitate fabrication of smart and efficient devices.^14^ Development and understanding of such interfaces are critical to develop biosensors on chips,^15^ and integrated microfluidic platforms for rapid and low-cost detection of genetic biomarkers.^16,17^

With all the growth in this research area, there is a constant need to review new developments. This review is distinct from DNA-based nanostructures (*e.g.* tetrahedral or origamis), which have been constantly reviewed.^18–22^ In the last five years, the reviews of DNA interfaces have covered only specific types of materials (*e.g.* gold, graphene, carbon nanotubes *etc.*)^23–26^ or a category of materials (*e.g.* 2D materials, nanoparticles).^27–29^ Here, we are reviewing all types of dimensional interfaces of the nanomaterials, except macroscopic interfaces (*e.g.* surface-assemblies on microbeads and macro electrode surfaces),^30–32^ with key developments in the biosensing applications of the DNA–materials interfaces since 2015.

### 1.1. 0D interfaces

Zero-dimensional materials represent a class of nanomaterials with all dimensions in nanometer scale in spherical or crystal shapes, *e.g.* quantum dots, nanoparticles, nanoclusters *etc.*^33^ The 0D materials have gained the major interest since the beginning of nanotechnology in the 1980s due to their powerful electronic, magnetic, and optical properties for broad applications. They differ incredibly in their properties due to their chemical makeup, size, shape, and environment. There is a plethora of applications associated with 0D materials from solar cells to drug delivery. It would be out of the scope of this review to list them all. Three major types of DNA–0D interfaces are discussed in this review because DNA interfaces with other types, *e.g.* polymer, metal–organic frameworks, are in their beginning stages.

#### 1.1.1. Metal nanoparticles interfaces

Metals interact with nucleic acids through various sites, and the interaction has great impact on the structure, conformation, stability, and electronic properties of DNA.^1^ Conjugation of DNA with metal nanoparticles is an interplay of electrostatic and van der Waals interactions between them.^34^ Farkhari *et al.* has recently explained the adsorption mechanism of ssDNA and dsDNA on gold nanoparticles (AuNPs) and silver nanoparticles (AgNPs) using surface enhanced Raman spectroscopy (SERS).^34^ They reported a higher affinity between DNA and AgNPs than the affinity between DNA and AuNPs. They proposed that each nucleobase interacts through van der Waals interactions from multiple sites involving N-atom of imine groups and ketonic oxygen atoms. There has been a paradox around adsorption propensity of dsDNA on AuNPs, explaining it through either electrostatic^35^ or hydrophobic^36^ interactions. However, the experimental results from Farkhari *et al.* found no difference in adsorption affinity of ssDNA and dsDNA from the same sequence type and suggested that the adsorption affinity of dsDNA is affected by its chemical environment, *e.g.* salt concentration^34^ and charge on cationic metal nanoparticles.^37^ The reported binding affinities have differed depending on the type of metal nanoparticles, DNA sequence, surface chemistry, and experimental conditions. The generally accepted binding affinity between the nucleobases and AuNPs is A > G > C > T.^38^ Therefore, blocks of adenine bases with probe sequences have been used to anchor probes on the nanoparticles,^39^ where the length of adenine blocks can be used to tune surface density as well as prevent non-specific adsorption on the surface. Modifications of the oligonucleotides at the 5′- or 3′-end *via* NH_2_–, SH– groups allow surface immobilization of the strands using EDC/NHS chemistry and Au–S linkage, respectively. Conjugation of AuNPs with (–SH) thiol modified oligonucleotides has been a popular method to form directed DNA–AuNPs interfaces for biomedical applications.^40^ However, there has been concerns in the scientific community about the exact nature of the Au–S linkage.^41^ Despite the debate, this linkage is by and large the most common conjugation method between the two materials. Modification of the DNA backbone with a phosphorothioate (PS–) group is also a significant adsorption strategy through simultaneous Au–S and Au–O bonding per nucleotide with adsorption ranking of thiol > PS > adenine > thymine.^42^

**Fig. 2:**
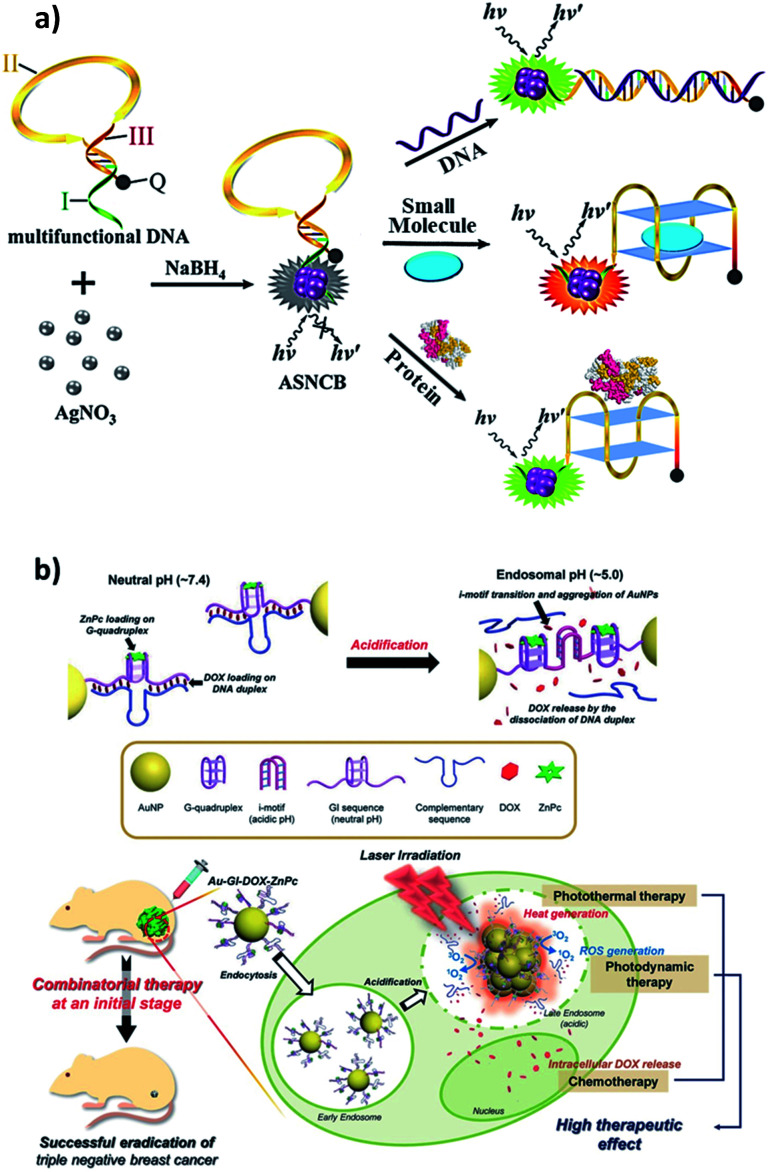
(a) Multifunctional activatable silver nanoclusters beacon (ASNCB) designed by interfacing single-stranded DNA sequences with silver for multiplex DNAs, small molecule, and protein sensing. Reprinted with permission from ref. 43 with copyright (2017) American Chemical Society. (b) Above, design of the Au-GI nanomachine and its operation mechanism depending on pH. Below, intracellular dynamic operation of the Au-GI nanomachine facilitating triple combination of photothermal, photodynamic, and chemotherapy^44^ with copyright (2018) Wiley and Sons.

Liu *et al.* leveraged on van der Waals interaction between DNA nucleobases and AgNPs to form nucleic acid-stabilized silver nanoclusters and designed a multipurpose molecular beacon probe so called ‘activatable silver nanoclusters beacon’ (ASNCB), as shown in Fig. 2a.^43^ It relies on target recognition induced conformational transition of the probe and yields fluorescent signal of silver nanoclusters. With slight variation in the probe, they were able to detect influenza A virus genes, ATP, and thrombin protein. By further implementing two different color ASNCBs, the ASNCB probe was implemented for ATP imaging in living cells. Park *et al.* developed functional DNA-decorated Au nanomachines for triple combinatorial anti-tumor therapy, which exploited photodynamic and photothermal properties of G-quadruplex and i-motifs modified Au nanoparticles (Au-GI) shown in Fig. 2b.^44^ Specifically, an anti-cancerous drug doxorubicin (DOX) and a zinc phthalocyanine photosensitizer (ZnPc) were loaded onto the i-motif/cDNA duplex and G-quadruplex, respectively. When the Au-GI was internalized into cancer cells of triple negative breast cancer (TNBC) tumor model, i-motif structure constituted between the nearby Au-GIs due to the acidic environment in the cells causing aggregation of Au-GIs leading to heat generation under NIR irradiation, thus photothermal ablation of cancer cells. While simultaneously the loaded DOX is released from i-motif/cDNA duplex because of the dehybridization. The photosensitizer (ZnPc) in the G-quadruplex produces singlet oxygen under illumination by 660 nm light, contributing to the photodynamic therapy.

#### 1.1.2. Metal oxide nanoparticles (MONPs) interfaces

Liu and coworkers have comprehensively investigated the DNA–MONPs interfaces.^45–50^ The interaction mechanism in general involves coordination of the phosphate backbone of DNA with MONP surface. Moreover, cationic surfaces also interact through electrostatic attraction, while anionic surfaces resist the adsorption due to repulsion. It was found that the adsorption process is normally independent of the type of nucleotides but affected by several other factors, such as DNA length and conformation, type of metal, charge on MONP, pH, and salt concentration. Shorter sequences adsorb with high density but lower stability, while lower adsorption affinity of dsDNA compared to ssDNA was exploited to detect hybridization events on MONPs surfaces through recovery of quenched fluorescence. Liu *et al.* has recently shown an interesting property of DNA called Janus character, where ssDNA orthogonally interacts with 2D graphene oxide (GO) through nucleobases and with MONPs through phosphate backbone.^49^ Although they report DNA adsorption affinity as CoO > NiO > Cr_2_O_3_ > Fe_2_O_3_ > Fe_3_O_4_ > TiO_2_ > CeO_2_, one would observe a different trend based on different experimental conditions due to the variable nature of metal oxides. Several proof-of-concept applications have been shown in the studies above using DNA–MONPs interactions, such as DNA hybridization detection, detection of metal ions, and detection of anions based on the displacement of the adsorbed ssDNA in presence of the analyte species.

#### 1.1.3. Quantum dots interfaces

Quantum dots (QDs) are semiconductor nanocrystals, traditionally comprised of chalcogenides and heavy metals, with unique size-dependent photoelectric properties. Interfacing chemistry between QDs (*e.g.* ZnS, CdS, or CdSe/ZnS core/shell nanocrystals) with DNA is by and large similar to Au–S linkage due to the high affinity between heavy metal atoms and the thiol group. Several sulfur-based compounds (*e.g.* alkyl-thiols, thiol-alkyl acids, disulfide containing cystine *etc.*) were explored to link DNA with the QDs.^51,52^ There could be other methods of linkage, such as physisorption or biomolecules such poly(histidine), streptavidin, and biotin *etc.*, nevertheless thiol linkage is a widely popular method of conjugation. QDs have remarkable photoluminescence properties that can be affected by size and conformation of the fluorophore labelled DNA.

**Fig. 3:**
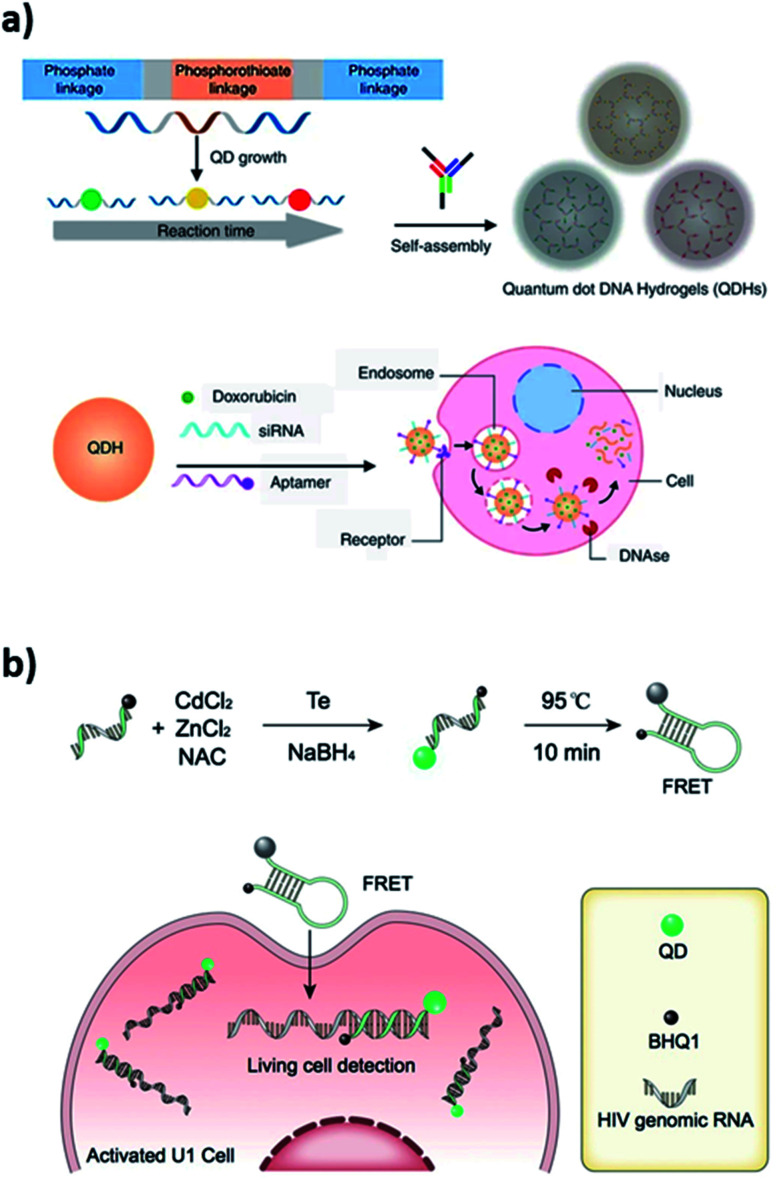
(a) Self-assembled quantum dot DNA hydrogels (QDHs) modified for cell-specific targeting with an aptamer and siRNA/drug delivery. Reprinted from ref. 53 with permission from Springer Nature. (b) Quantum dots nanobeacons (QD-NBs) comprising DNA hairpin structure for single RNA labelling and imaging in live cells. Reprinted with permission from ref. 54 with copyright (2019) American Chemical Society.

DNA–QD interfaces are unique interfaces that allow DNA-templated conjugated systems to have a wide range of applications. Recently, a novel application illustrated in Fig. 3a involved a single step incorporation of DNA-templated quantum dots (ZnS–QDH) with high quantum yield, long-term photostability and low cytotoxicity into a hydrogel network. These quantum dot DNA hydrogels were used for delivery of an anticancer drug, doxorubicin, into MDA-MA-231/Luc breast cancer cells implanted into mice. The drug delivery by Dox–ZnS–QDH increased the drug efficacy by 9-fold.^53^ Ma *et al.* developed a series of quantum dots nanobeacons (QD-NBs) for single RNA labelling and imaging in live cells.^54^ The QD-NBs were synthesized with controllable 1–4 valencies by conjugating a black hole quencher (BHQ1) and phosphorothioate comodified DNA onto CdTe:Zn^2+^ QDs *via* a one-pot hydrothermal method. A QD-NB with one conjugate DNA was proven to be suitable and effective for imaging single HIV-1 RNA, where target nucleic acid sequences were hybridized with the stem-loop hairpin DNA and recovered the QD fluorescence for ultrasensitive detection in live HIV-1 integrated cells (Fig. 3b).

### 1.2. 1D interface

One-dimensional or 1D materials are classified as nanomaterials having two dimensions at the nanoscale, while their third dimension is more than 100 nm. The geometric shapes proposed for these nanomaterials include nanotubes, nanofibers, nanobelts, nanorods, and nanowires as illustrated in Fig. 1a. Carbon nanotubes (CNTs) were among the most studied 1D nanomaterial when interfaced with DNA.^55^ However, several other types of interfaces with 1D nanostructures were developed, such as halloysite clay nanotubes (HNTs)^56^ and metal nanowires (*e.g.* silicon nanowires, SiNW).^57^

#### 1.2.1. Carbon nanotubes (CNTs) interfaces

**Fig. 4:**
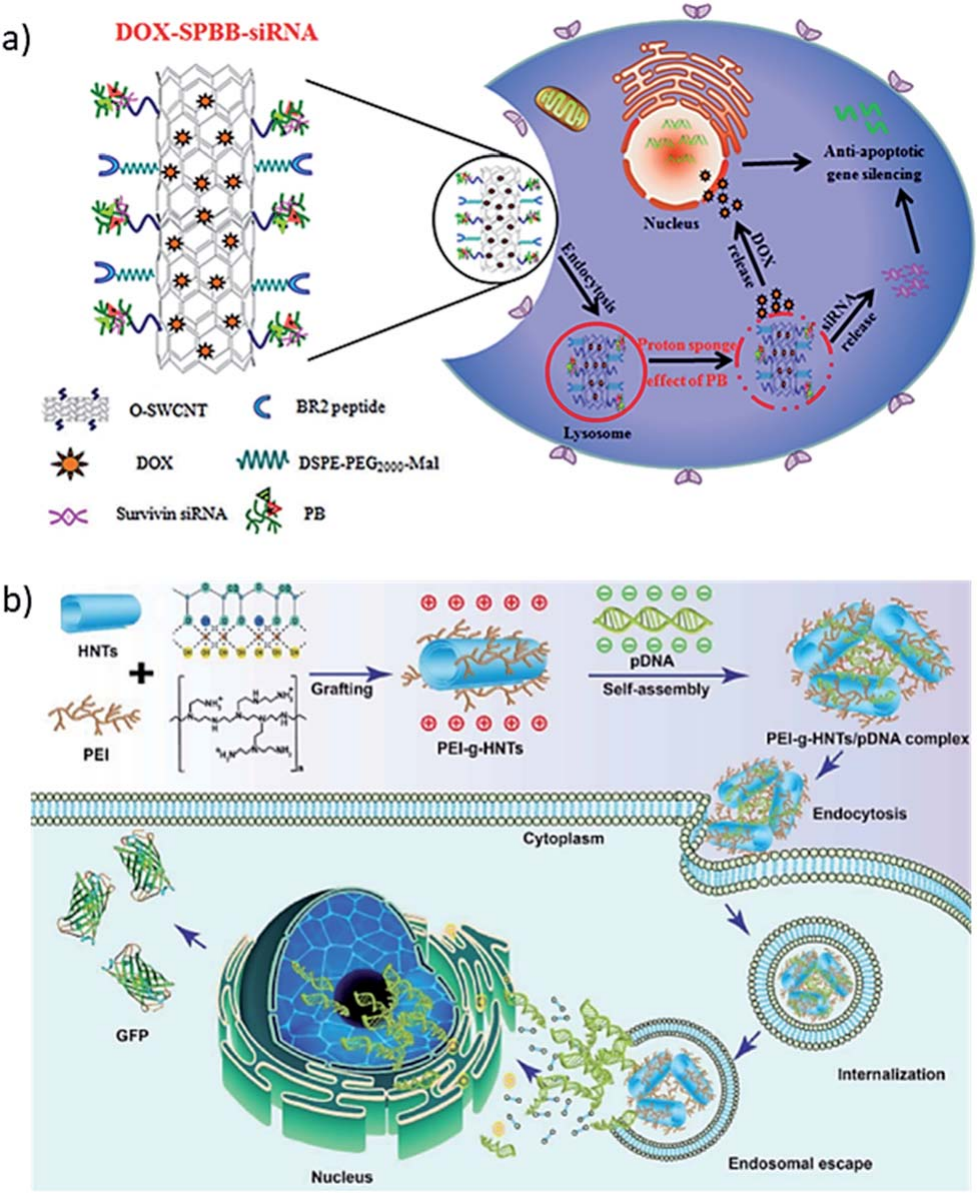
(a) Synthesis process for DOX–SPBB–siRNA, a conjugate of single-walled carbon nanotubes loaded with doxorubicin and survivin RNA, which release the drug and the RNA inside the tumor cell. Reprinted with permission from ref. 64 with copyright (2019) American Chemical Society. (b) Polyethyleneimine (PEI) grafted HNTs bind green fluorescence protein (GFP) labeled pDNA by electrostatic interaction. The PEI-*g*-HNTs/pDNA complex was taken by cancer cells and show higher transfection efficiency towards both 293T and HeLa cells. Reprinted with permission from ref. 72 with copyright (2017) Elsevier.

The physisorption of DNA on the surface of neutral CNT is driven by π–π stacking with a binding affinity of A > G > T > C,^58^ which allows the negatively charged phosphate backbone to disperse the DNA–CNT conjugate in aqueous medium.^55^ The DNA wrapping around the CNTs through π–π stacking is further strengthened by H-bonds between the wrapping nucleobases, while high ionic strength increases the DNA surface coverage around the nanotubes.^59^ Moreover, both can be conjugated through covalent attachment, where carboxylic functionalized CNTs can be attached with amine functionalized DNA using EDC/NHS chemistry.^60^ There are other methods of conjugation such as functionalized pyrene for π–π stacking with CNTs,^61^ and functionalized phospholipids to interact with CNT through van der Waals forces.^62^ There is a plethora of reports on electrical and electrochemical DNA biosensing applications using CNT-based electrode surfaces due to the superior charge transfer properties of CNTs.^63^ CNT-based gene delivery has been explored for almost two decades. In an interesting report, Cao *et al.* designed a system for the codelivery of an anticancer drug and a small interfering RNA. They synthesized a pH-responsive, surface-modified single-walled carbon nanotube (SWNTs) for the codelivery of doxorubicin (DOX) and survivin small interfering RNA (siRNA).^64^ In this example, polyethyleneimine (PEI) covalently conjugated with betaine was interacted with oxidized SWCNT to form SWCNT-PB (SPB). The SPB complex had pH-responsive lysosomal escape ability. The DOX and survivin siRNA were noncovalently adsorbed onto SPB conjugate, which was further modified with the targeting and penetrating peptide BR2 to form DOX–SPBB–siRNA (Fig. 4a). As a result, they achieved a considerably higher uptake of siRNA. Furthermore, siRNA/DOX was released into the cytoplasm and nuclei of adenocarcinomic human alveolar basal epithelial (A549) cells without lysosomal retention. DOX–SPBB–siRNA was reported to significantly reduce tumor volume in A549 cell-bearing nude mice, demonstrating the synergistic effects of DOX and survivin siRNA.

#### 1.2.2. Halloysite nanotubes (HNTs) interfaces

Halloysite is aluminum silicate, Al_2_Si_2_O_5_(OH)_4_·*n*H_2_O, clay mineral with nanotubular structure.^65^ HNTs have a variety of biomedical applications, such as controlled drug release,^66,67^ gene delivery,^68^ capture of tumor cells,^69^ and tissue engineering.^70^ Interaction of DNA with HNTs was first studied through forced solid-state mixing and attributed their conjugation to π–π interaction between the DNA backbone and HNTs.^56^ The π–π interaction between the two materials can improve in presence of Mg^2+^ ions by reducing repulsion between negatively charged HNTs and the DNA phosphate backbone.^71^ However, there were also reports of polymer mediated interactions (*e.g.* polyethyleneimine or PEI, γ-aminopropyltriethoxysilane or APTES)^68,72,73^ for gene delivery with a non-viral gene vector. In such example, Long *et al.* used PEI grafted HNTs (PEI-*g*-HNTs) using APTES as a bifunctional linker as illustrated in Fig. 4b.^72^ The positively charged PEI-*g*-HNTs adduct was bound to DNA through electrostatic interaction with a N/P ratio from 5 : 1 to 40 : 1 to form PEI-*g*-HNTs/pDNA complexes. The PEI-*g*-HNTs/pDNA complex showed a higher transfection efficiency of up to 46.8% towards 293T and HeLa cells compared to a 41.6% efficiency of the experimental control, which translated into 32% higher green fluorescence protein (GFP) expression in the cells because of DNA release from the PEI-*g*-HNTs/pDNA complex.

### 1.3. 2D interface

Two-dimensional or 2D materials represent a class of nanomaterials having a thickness of a few nanometers or less, while their other two dimensions are more than 100 nm. They are also considered as crystalline solids comprising a single layer of atoms, but most of the studies do not follow this strict definition to explain their DNA–2D interfaces. The geometric shapes proposed for these nanomaterials include nanosheets, nanofilms, nanoflakes, and nanolayers as shown in Fig. 1a. Electrons in 2D materials are free to move in the two-dimensional plane, and the electronic structure changes with the thickness, which can be harnessed for biosensing signaling. The eminent examples of these materials are graphene and transition metal dichalcogenides (TMDs), which have been extensively interfaced with DNA among other 2D materials.

#### 1.3.1. Graphene interfaces

**Fig. 5:**
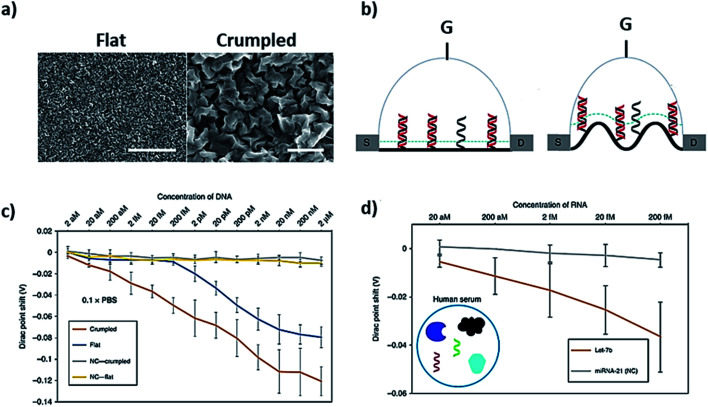
Crumpled graphene FET DNA biosensor. (a) SEM images of flat and crumpled graphene, (b) field-effect transistor made of flat and crumpled graphene to detect DNA, (c) shift in Dirac potential because of DNA hybridization, and (d) detection of DNA in human serum using G-FET. Reprinted with permission from ref. 78 with copyright (2020) Springer Nature.

Molecular dynamic (MD) simulation was used to explain the dynamic process of the adsorption of ssDNA and dsDNA onto pristine graphene.^74^ They proposed that the adsorption kinetics are driven by the π–π stacking interaction between DNA and pristine graphene. The DNA interfaces with pristine graphene have been explored using field-effect transistors known as G-FET.^75–78^ Dontschuk *et al.* noted change in charge carrier density for the adsorption of an individual nucleobase on G-FET channel.^75^ They attributed the shift nucleobase-specific with a trend of G < C < T < A, which is the effect of molecular adsorption of an individual nucleobase on the electronic structure of graphene. Several studies used a 1-pyrenebutanoic acid succinimidyl ester (PASE) linker to immobilize DNA on the graphene surface. The PASE linker binds to graphene by the π stacking of its pyrene group, while the succinimide portion of PASE extends out from the surface to bind 5′-amine-modified probe DNA. Xu *et al.* used the PASE immobilization strategy to study the kinetics of DNA hybridization on a multi-channel G-FET by patterning graphene single-crystal domain in an array format.^77^ When DNA hybridization occurred on the surface, it caused variations in charge at the interface, leading to variations in electrostatic potential in the graphene channel and positive shifts. The device achieved a detection limit of 10 pM for DNA and could detect a single-base mutation quantitatively in real time, while the sensor chip could be regenerated more than 50 times with >90% functional recovery. Hwang *et al.* fabricated the FET channels with deformed monolayer graphene (crumpled) for the detection of nucleic acids as shown in Fig. 5a and b.^78^ They claimed to achieve a 10 000 times better sensitivity through measuring the shift in Dirac potential with crumpled graphene channels with 600 zM in buffer and 20 aM in human serum, which are ∼18 and ∼600 nucleic acid molecules respectively (Fig. 5c and d). There are other derivatives of graphene, *i.e.* graphene oxide (GO) and reduced graphene oxide (rGO), which have also been studied in detail for interfacing DNA. We are not reviewing those derivatives as they have been reviewed a number of times elsewhere.^25^

#### 1.3.2. MoS_2_ interfaces

In recent years, transition metal dichalcogenides (TMDs) were extensively explored for DNA biosensing applications employing a variety of detection systems. TMDs comprise of a transition metal atom (*e.g.* Mo, W) bound with two chalcogen atoms (*e.g.* S, Se or Te). TMDs exhibit a unique combination of atomic-scale thickness, direct bandgap, and good electronic properties, which make them attractive for a variety of high-end electronics and biosensing applications.^79^ Among TMDs, MoS_2_ is the most studied material to explain the interaction mechanism between the TMDs and DNA. MD simulations showed that dsDNA adsorbs on an MoS_2_ surface in stand-up orientation through terminal bases driven by weak van der Waals force, which suggests that the adsorption of DNA on TMDs is weaker than GO surfaces. It was also suggested that the interaction does not result in destabilization of the dsDNA structure.^80^ Theoretical and experimental studies indicate that MoS_2_ has the highest adsorption affinity for guanine bases.^81–83^ Moreover, it was also found that longer DNA sequences may show better adsorption on MoS_2_ surface, which can improve the stability of the DNA/MoS_2_ conjugate. Such sequence- and length-dependent adsorption affinities were used to design biosensing surfaces on TMDs using diblock probes, where homonucleotide tails helped the immobilization of a molecular beacon probe with a desired surface density.^84^

**Fig. 6:**
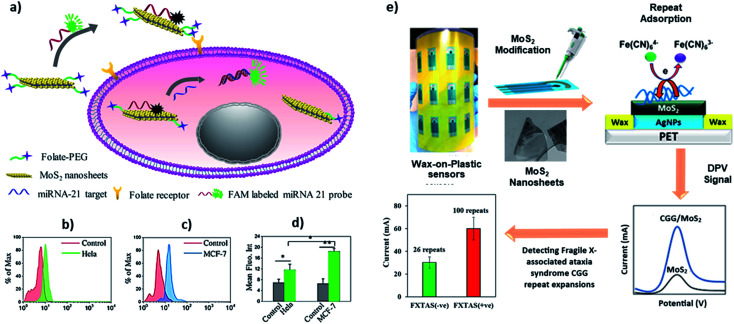
(a–d) Interface of ssDNA–MoS_2_–PEG–FA probe-based FRET platform for intracellular miRNA-21 detection in cancer cells, *i.e.* HeLa and MCF-7 cells. Reprinted with permission from ref. 85 with copyright (2018) American Chemical Society. (e) Label-free electrochemical detection of fragile X-associated repeats (CGG repeats) on MoS_2_ nanosheets modified wax-on-plastic platforms. Reprinted with permission from ref. 86 with copyright (2020) American Chemical Society.

MoS_2_ is a good fluorescence quencher and has excellent electron transport properties, which allows a simple DNA biosensing application employing labelled and label-free strategies. Oudeng *et al.* demonstrated an interesting application of one-step *in situ* detection of targeted miRNAs expression in single living cancer cells *via* MoS_2_ nanosheet-based fluorescence on/off probes, Fig. 6a.^85^ In this application, probe ssDNA/MoS_2_ nanosheets were functionalized folic acid (FA)–poly(ethylene glycol). The folic acid receptors on cancer cells facilitated internalization of the probe ssDNA/MoS_2_ nanosheets, and the hybridization between the probes and target miRNA-21 in MCF-7 and HeLa cells caused the detection of green fluorescence following detachment of the formed duplex (Fig. 6b–d). Such non-toxic probes can potentially provide a real-time, one-step detection system for disease-relevant intracellular miRNAs. Recently, our group has demonstrated a label-free application of DNA/MoS_2_ system for flexible and highly sensitive electrochemical sensors dubbed as “wax-on-plastic” platforms as shown in Fig. 6e.^86^ These platforms can be easily fabricated through desktop wax and inkjet printers.^87–89^ Specifically, it was demonstrated that physisorbed DNA improves the electrochemical property of MoS_2_ electrodes, because it reduces the bandgap of the MoS_2_. The electrocatalytic current is sequence-dependent, and the hybridization event further enhances the response in the high ionic strength environment, which was translated into ultra-sensitive detection of CGG trinucleotide repeats associated with fragile X–associated tremor/ataxia syndrome. The detection limit of the platform for the prehybridized CGG duplexes was 0.4 aM and was 0.1 pM for the surface hybridization event.

### 1.4. 3D interface

Three-dimensional (3D) materials as depicted in Fig. 1a represent a type of materials having 3D shape with overall dimensions of more than 100 nm. This class is in between macroscale and nanoscale because these materials are outgrowths of nanostructures. They can be found in a wide range of morphologies, such as dendrites, coils, hollow spheres, cubes, spindles, pillars, and nanoflowers.^90–93^ The performance and applications of these materials rely on their sizes, shapes, dimensions, and morphologies. For instance, due to their morphology, they can provide enough adsorption sites for involved molecules, while three dimensional access to the surface can improve mass transport of the molecules (*e.g.* probe and target).^94–96^ Therefore, 3D structured sensing electrodes allow a high surface area to immobilize a probe and a high access to target diffusion resulting in extremely low detection limits in real samples.^97^ Chemically, 3D structures can be comprised of metal-based,^98^ carbon-based, or polymer-based^99^ materials. The interfacing mechanisms to immobilize DNA on 3D materials can be either an adsorption or chemisorption mechanism depending on the chemical makeup of the materials. For example, graphene-based composites can interact through electrostatic attraction and strong π–π stacking with the DNA phosphate backbone and nucleobases.^100^ 3D structures can also be functionalized with different groups to immobilize biological probes. For instance, perylene tetracarboxylic acid (PTCA) functionalized graphene surface involves π–π interaction and hydrophobic forces between graphene and perylene moiety, while providing active sites for immobilization of the 5′-NH_2_ modified probe DNA.^101^ On gold 3D structures, Au–S chemistry can be used to immobilize DNA like metallic nanoparticles.^97^

**Fig. 7:**
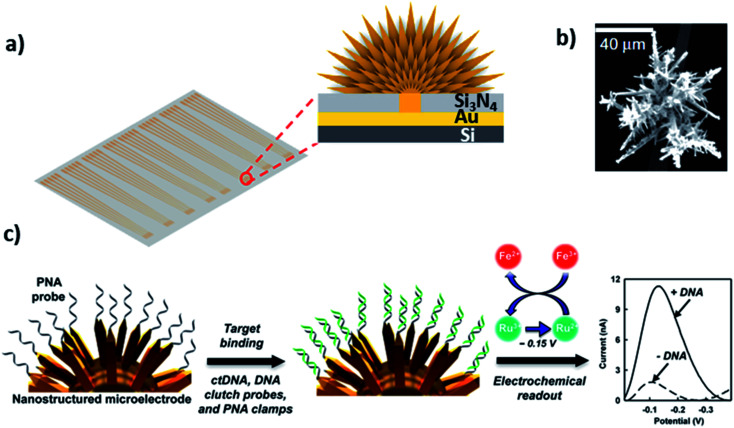
(a and b) 3D structured gold electrode so-called “nanostructured microelectrodes or NMEs” grown on silicon chip and scanning electron microscopic image of 3D structured gold electrode. Reprinted with permission from ref. 102 with copyright (2015) Springer Nature. (c) PNA probe immobilized on gold 3D structures by Au–S linkage, which allows ultrasensitive electrochemical detection of circulating tumor DNA in human serum. Reprinted with permission from ref. 103 with copyright (2016) American Chemical Society.

Kelley and coworkers developed the so-called 3D nanostructured microelectrodes (NMEs) by growing 3D structures on gold microelectrode surfaces for electrochemical nucleic acid detection (Fig. 7a).^97,102^ These sensing electrodes offer a large surface area (Fig. 7b), which improves sample diffusion to electrode surface, facilitating binding with surface bound probe and resulting in extremely low detection limits in biological samples. For example, chip-based 3D NMEs were applied for electrochemical clamp assay for direct, rapid analysis of circulating nucleic acids in serum, Fig. 7c. Specifically, they immobilized peptide nucleic acid probes (PNA) *via* Au–S chemistry on the NMEs and monitored differential pulse voltammetry (DPV) response before and after exposure to the target in serum. The clamp approach before the actual measurement blocks the non-specific strands and only allows the mutated sequence to bind the surface-bound probe. The presence of mutations can be detected within 15 minutes with a limit of detection of 1 fg μL^−1^ and dynamic range from 1 fg μL^−1^ to 100 pg μL^−1^.^102^ They also found that NMEs with clutch probe assay exhibited excellent sensitivity and specificity in the detection of mutated circulatory tumor DNA (ctDNA) with detection sensitivity of 1 fg μL^−1^ of a target mutation in the presence of 100 pg μL^−1^ of wild-type DNA, approaching detecting mutations at a level of 0.01% relative to wild type.^103^

## 2 Conclusion

We have reviewed here the diverse class of interfacial nanomaterials comprising DNA and dimensional nanomaterials. DNA can be directly interfaced with a range of 0D, 1D, 2D, and 3D nanomaterials through physisorption and chemisorption where the interactions induce new interfacial character to the materials. For each type of these materials, we discussed the possible and popular interfacing strategies followed by some key recent applications. Unique conformational properties and nucleobase specificity of DNA along with its ability to interact with other materials chemically and through pi-stacking, van der Waals forces, and electrostatic interactions are the primary driving forces behind the novel biomedical applications. These applications—gene therapy, drug delivery, photodynamic therapy, cell imaging, multidimensional biosensing—depend on the structural changes in the DNA triggered by its target as well as the signaling properties of the dimensional materials. Current developments in this area are pushing the boundaries and proposing applications which are specific, sensitive, and applicable for clinical use.

## 3 Future outlook

The rapid growth in DNA–dimensional nanomaterials have opened new avenues for novel biomedical research. The biocompatibility of such materials promises their use for noninvasive surgical treatment through photothermal and photodynamic properties such as DNA–0D system, *e.g.* Au-GI nanomachines. While gene therapy and chemotherapy would be performed simultaneously in the future using DNA–1D interfaces, such as halloysite clay nanotubes which are inexpensive and abundant in nature. Moreover, excellent electrical and electrochemical properties of 2D graphene and MoS_2_ nanosheets are available to fabricate low-cost miniaturized DNA biosensing devices for point-of-care diagnostics, for *e.g.* wax-on-plastic flexible sensors. Nevertheless, the PNA–3D nanostructured microelectrodes are close to clinical application of the detection of genetic biomarkers in serum with high sensitivity. The field is anticipated to flourish with hybrid systems involving DNA interfacing with multiple dimensional materials directly or orthogonally for multidimensional applications.

## Authors contribution

NA contributed to conceptualization, investigation, validation, and writing original draft. PKD and AHO contributed to investigation. GD contributed to writing review and editing. MHS contributed to funding acquisition, supervision, writing original draft, editing, and reviewing.

## Permission note

Authors confirm that permissions have been received for the figures used in this article.

## Conflicts of interest

Authors claim no conflict of financial or intellectual conflict of interest.

## Supplementary Material
